# Bridging the gap: *Prevotella/Segatella's* impact on gut barrier function and advanced cultivation strategies to realize the uses in gut health

**DOI:** 10.1080/19490976.2026.2638001

**Published:** 2026-03-02

**Authors:** Shuang Wang, Tao Zhou, Xiuqi Wang, Jiangchao Zhao, Xiaofan Wang

**Affiliations:** a College of Animal Science, South China Agricultural University, State Key Laboratory of Swine and Poultry Breeding Industry, National Engineering Research Center for Breeding Swine Industry, Guangdong Laboratory for Lingnan Modern Agriculture, Guangdong Provincial Key Laboratory of Animal Nutrition Control, Guangzhou, People's Republic of China

**Keywords:** Culturomics, intestinal barrier function, personalized microbial therapeutics, *Prevotella*, *Segatella*

## Abstract

*Prevotella* and *Segatella* are important, keystone genera in the gut microbiota, renowned for their exceptional fiber-degrading capacity. These genera critically modulate gut microbial composition, influence host metabolic pathways and gut barrier function, and exhibit formidable ecological niche competitiveness, underscoring their pivotal role in gut ecosystem dynamics. While they dominate healthy gut microbiomes, their probiotic potential on epithelial barrier function has been disproportionately overlooked. This review comprehensively elucidates their microbial eco-profiling and the underlying molecular mechanisms in sustaining intestinal barrier function, considering physical, chemical, biological, and microbiological dimensions, thereby providing insights relevant to the prevention and treatment of intestinal diseases such as inflammatory bowel disease, irritable bowel syndrome, and metabolic disorders. Most importantly, we have summarized 23 current commercial and research-based isolation and cultivation approaches for *Prevotella*/*Segatella*, integrating the emerging high-throughput methodologies to expand the available strain repertoire. We also emphasize the critical need for subsequent research to characterize strain-specific functional profiles through multi-omics approaches, which will be essential for developing targeted and personalized microbial therapeutics.

## Introduction

*Prevotella* emerged as a key player in host-gut microbiome interactions following the seminal work of gut microbiome enterotyping in 2011^1^. For a long time, the metabolic characteristics of human health-related microbes were thought to be clearly outlined based on the composition of the intestinal microbiomes, leading to the concept of “enterotype”.^1^ The dominant bacterial taxon and its symbiotic partners within a given enterotype exhibit complementary metabolic functions and physiological traits, demonstrating both functional robustness and environmental tolerance. Pioneering cohort studies revealed that industrialized populations consuming high-fat, high-protein diets typically harbor *Bacteroides*-dominated (B-type) microbiomes linked to their robust energy harvest capability. In contrast, non-industrialized regions with fiber-rich diets predominantly sustain *Prevotella*-dominated (*P*-type) communities.^2‐4^ Moreover, compared to the B-type, which are highly associated with gut microbiota imbalance, low diversity, virulence factors and antibiotic resistance genes, and metabolic diseases such as cardiovascular disease and diabetes,^5‐10^ their *P*-type-counterparts promote better gut health, including increased diversity of genera, production of short-chain fatty acids (SCFAs), lowering of the threshold of immune stress, maintaining host health and longevity and reduction of the gut transit time in relation to bowel habits such as constipation.^11‐19^ Although lateral studies have suggested that the generalization of enterotypes may be overly simplistic, the role of *Prevotella* in breaking down plant fibers to create a beneficial barrier function in the gut is becoming increasingly clear.^20^

The *Prevotella* (reclassified into seven genera, including *Prevotella* and *Segatella*) genus,^21^ as a dominant gut commensal, critically regulates the intestinal mucosal barrier through multiple mechanisms. Primarily, these bacteria enhance the intestinal physical barrier by modulating key junctional complex proteins, including tight junctions (TJs), adherens junctions (AJs), and desmosomal bridging granules.^22^^,^^23^ Then, they enhance the chemical barrier through the production of SCFAs and bacteriocins.^24^ Additionally, some of these bacteria actively modulate gut immune homeostasis through direct interactions with various immune cells and cytokines to maintain mucosal and systemic equilibrium.^25^^,^^26^ Furthermore, they orchestrate gut defense by establishing a competitive biological barrier through niche exclusion and nutrient depletion, effectively outcompeting pathogens.^27^ Given these properties, dietary interventions targeting their enrichment, such as supplementation with fermentable carbohydrates like isomaltooligosaccharides (IMOs), resistant starches, and inulin, are increasingly advocated for improving gut health globally.^28‐30^

However, a striking paradox exists although *Prevotella* and *Segatella* play a crucial role in host health, they are not currently recognized as direct-fed microbes. No countries or regions, including the United States, Europe, or Asia, have included it on their lists of approved edible additives. This translational gap stems from two interrelated fundamental bottlenecks. First, there is a critical scarcity of culturable isolates, hindering rigorous safety and efficacy testing. Second, their ecological and health impacts are profoundly strain- and context-dependent, moving beyond simple beneficial or harmful dichotomies. This review, therefore, aims to navigate this paradox through a structured examination. First, it elucidates the genomic and functional complexity that underpins the diverse, context-dependent roles in barrier protection, which establishes the central challenge of precision. Second, we detail the innovative, genomics-driven cultivation strategies that are currently overcoming the isolation barrier, offering practical pathways for strain recovery. Finally, we examine the challenges and frameworks that must be addressed to achieve their safe and precise therapeutic application.

## Taxonomic delineation and ecological associations

**Figure 1.**
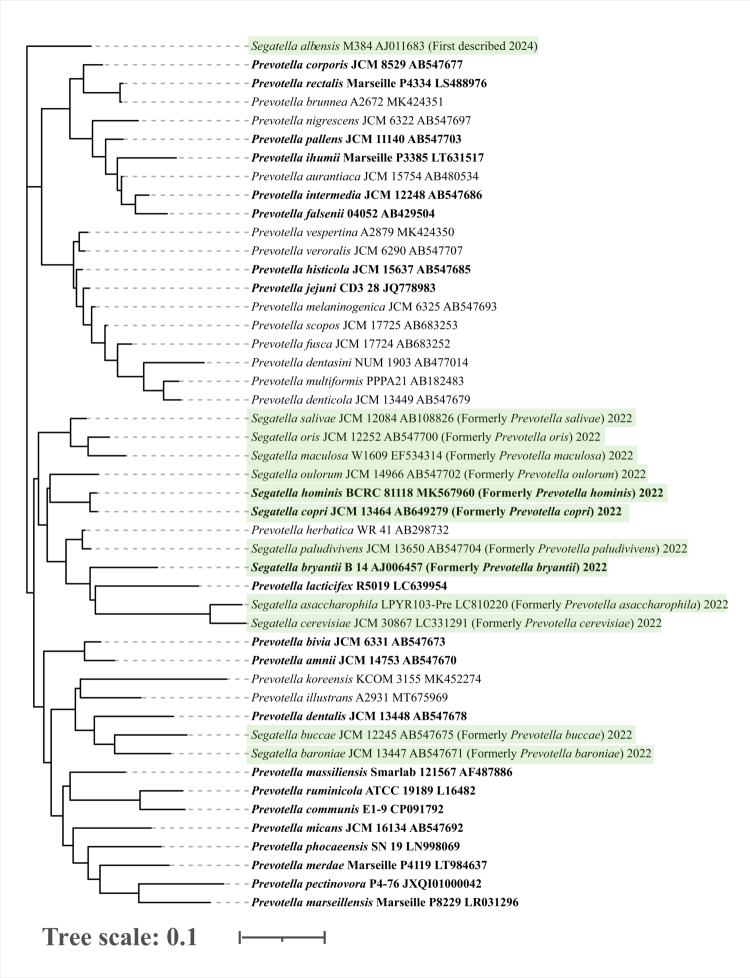
Phylogenetic tree of 47 *Prevotella* and *Segatella* strains covering all species, except *P. genomosp*. *C1*, *P. genomosp*. *C2*, *P. genomosp. P6*, *P. genomosp. P4*, *P. conceptionensis*, *P. ihumii*, Candidatus *P. avicola*, Candidatus *P. intestinigallinarum*, Candidatus *P. stercoripullorum*, Candidatus *P. equi*. Strains reclassified as *Segatella* from *Prevotella* are highlighted in green. Gut-dominant *Prevotella* species are shown in bold. Tree visualization was performed with iTOL.

*Prevotella* is a genus of Gram-negative, anaerobic, non-motile, non-spore-forming bacteria, and it was named by Shah and Collins in 1990 after the anaerobic microbiologist A. R. Prévot.^31^ In 1921, *Prevotella* was first isolated by Oliver and Wherry from multiple human organs, including the gut, vagina, respiratory tract, and oral cavity,^32^ and then was widely found in soil, animals, and other environment.^33^ As of May 2025, integrated metagenomic and culturomics approaches have successfully characterized over 50 bacterial species (Figure 1), such as *P. stercorea*, *P. intermedia*, *P. oralis*, *P. melaninogenica*, and *P. ruminicola*, with genomic data publicly available through NCBI. Among these species, *P. histicola*, *P. falsenii*, *P. pectinovora*, *P. ihumii*, *P. merdae*, *P. rara*, *P. loescheii*, *P. colorans*, *P. buccalis*, *S. bryantii*, *S. hominis*, *S. copri* were consistently identified as gut commensals. *S. copri*, formerly known as *P. copri*, was reclassified into the newly described genus *Segatella* in 2022. This revision is firmly supported by comparative genomic analyzes.^34^ The foundational study demonstrated that the Average Amino Acid Identity (AAI) between Clade 1 (the core *Prevotella* group containing the type species) and other clades, including the clade proposed as *Segatella* (e.g., Clade 3 in that study), was consistently below 70%, failing to meet the > 65% threshold for genus delineation.^35^ Furthermore, the Percentage of Conserved Proteins (POCP) only sporadically exceeded the 50% threshold between clades.^36^ These objective metrics provided the definitive genomic rationale for delineating these lineages into distinct genera, thereby justifying the separation of *Segatella* from *Prevotella*. Later in 2023, metagenomic analyzes expanded the *S. copri* complex to include 13 distinct species-level clades. These clades exhibit considerable genomic divergence, particularly in functional attributes such as carbohydrate-active enzyme (CAZyme) profiles, mucin utilization, bacteriocin synthesis, and antibiotic resistance genes (Figure 1),^21^^,^^34^^,^^37^ and these genomic distinctions may underpin opposing roles in intestinal barrier regulation.^27^^,^^37^^,^^38^ Certain clades specialized in dietary fiber fermentation likely enhance barrier function through increased production of SCFAs such as propionate, which has been shown to upregulate mucin (MUC2) expression,^39^^,^^40^ and to strengthen epithelial tight junction integrity.^41^^,^^42^ In contrast, clades enriched with mucin-degrading enzymes may impair epithelial barrier stability, thereby elucidating the dual associations of *S. copri* with both beneficial and detrimental health outcomes.^38^ Given these complex roles of *S. copri* in intestinal mucosal barrier function, the genus *Segatella* is included herein. For clarity, the taxonomic names used in the original literature have been retained. For definitive taxonomic reclassification, we advise consulting the primary literature to obtain the relevant microbial sequences before querying genomic databases.^21^

The taxonomic diversity and expansive genomes (up to 8.301 Mb) of *Prevotella* and *Segatella* underpin their profound ecological role as critical functional conduits linking dietary patterns, host physiology, and health status. This role is manifest in a distinct trajectory across the host lifespan.^43‐45^ In early life, as demonstrated in a study of infants from rural Gambia on a traditional high-fiber diet, *P. copri* and *P. stercorea* rapidly establish dominance in the gut microbiota post-weaning.^3^ Notably, a higher abundance of *P. stercorea* is directly associated with reduced frequency and duration of childhood infections, underscoring its function as a beneficial colonizer and health “facilitator”.^46^ Conversely, later in life, a marked decline in *Prevotella* abundance is widely observed in elderly populations. This attenuation correlates with key indicators of ecosystem frailty, including compromised intestinal barrier integrity, elevated systemic inflammation, and dysregulated metabolic homeostasis, signifying the erosion of its role as an ecological “maintenance factor”.^11,47‐50^ Consequently, the population dynamics of these genera collectively constitute a robust, lifespan-integrated functional biomarker for gut ecosystem robustness.^51‐53^ This dynamic, life-stage-associated biomarker is not a mere correlate; it is a functional readout of the gut ecosystem's state. The driver of this function, and the foundation of their ecological role, is their unparalleled capacity to degrade complex dietary fibers. This ability to shape the gut metabolic landscape forms the foundational mechanism through which *Prevotella*/*Segatella* influence the intestinal barrier. We now examine the genetic basis and enzymatic arsenal that enable this capacity, detailing their specific substrate preferences and how these metabolic strategies determine their competitive success and cooperative roles within the gut community.

## Metabolic preference and niche competition: laying the foundation for barrier reinforcement

**Figure 2.**
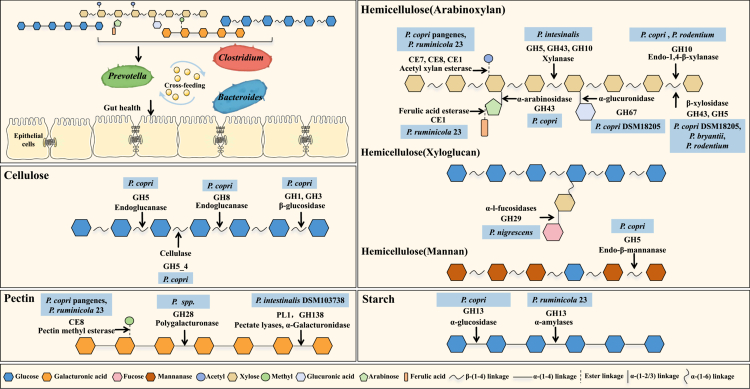
Schematic overview of dietary polysaccharide degradation by *Prevotella*. Highlighted taxa indicate specific *Prevotella* strains involved in degrading cellulose, hemicellulose, pectin, and starch via glycoside hydrolases (GH), carbohydrate esterases (CE), and polysaccharide lyases (PL). Enzyme families and substrate types are annotated.

*Prevotella* and *Segatella* are versatile gut bacterial members that possess over 100 CAZymes encompassing different families including glycoside hydrolases (GH), carbohydrate esterases (CE), and polysaccharide lyases (PL) that engage in wide degradations of various carbohydrates, such as starch, cellulose, hemicellulose, pectin, etc (Figure 2).^54^^,^^55^ Specifically, most species express genes encoding hemicellulose-degrading enzymes such as GH43, GH10, GH5, CE1, and CE6, followed by pectin-degrading enzymes like CE8, GH28, and PL1.^30,56‐59^ Notably, certain *Prevotella* members, including *P. copri*, *P. rodentium*, and *P. ruminicola*, produce cellulose-degrading enzymes such as CEL (GH5_4 family cellulase), Endoglucanase (GH8), and *β*-glucosidase (GH1).^55^^,^^56^ Among these, *P. copri*, a key commensal bacterium in the *P*. enterotype gut, exhibits extensive carbohydrate metabolic capabilities. It enhances glucose metabolism and insulin sensitivity via dietary fiber fermentation,^11^^,^^60^ modulates host serum metabolome and contributes to regulating systemic inflammation and glucose homeostasis.^60^^,^^61^ Among these diverse metabolic activities, the breakdown of hemicellulose is particularly critical, serving as the key interface between *Prevotella*'s enzymatic arsenal and its role in driving interspecies cooperation and competition within the gut ecosystem.^55^^,^^56^

Hemicellulose typically constitutes less than one-third of plant fiber components, together with cellulose, embedded within plant cell walls. A diverse consortium of microorganisms in humans and animals contributes to the degradation of hemicellulose including *Bacteroides ovatus*, *Bifidobacterium longum*, and *Butyrivibrio fibrisolvens*, etc. While *B. fibrisolvens* and *B. longum* are capable of degrading certain hemicellulose constituents such as xylans, they exhibit limited efficiency against other important polysaccharides like *β*-glucans and glucomannans.^44^^,^^62^^,^^63^ In contrast, *B. ovatus* effectively hydrolyzes specific hemicellulose subtypes, including xylan and galactomannan, via dedicated polysaccharide utilization loci (PULs) and enzymes such as glycoside hydrolase family 26 (GH26) *β*-mannanases.^64‐67^ However, its substrate range remains relatively narrow. Notably, *Prevotella* species, demonstrate the broadest and highly efficient hemicellulose-degrading capabilities and have been characterized as exceptionally proficient in the breakdown of diverse hemicellulosic substrates. *Prevotella*'s potent hemicellulolytic activity initiates a pivotal cascade in fiber digestion**:** by dismantling the outer hemicellulose wall, it exposes the underlying cellulose for access by specialized cellulolytic bacteria such as *Bacteroides* and *Clostridium.*^68^^,^^69^ This mechanistic role, creating a nutritional niche that drives a community shift, is evidenced by the rapid proliferation of *Prevotella* (up to 30% of total bacteria) following an abrupt dietary switch from sow milk to plant-based feed in piglets.^43^ The resulting consortium of fiber-degrading microbes collaboratively produces significant amounts of short-chain fatty acids (SCFAs), which contribute an additional 5-10% of energy to the host and play a crucial role in nourishing the intestinal barrier.^70^

Additionally, the CAZyme repertoire of *Prevotella* determines the outcome of interspecies competition through selective occupation of distinct and overlapping metabolic niches. For instance, among the three colonizable species (*P. intestinalis*, *P. rodentium*, and *P. muris*), *P. intestinalis* consistently outcompeted the rest overwhelmingly in two sequential co-housing mouse experiments (Figure 2). Genomic analysis revealed that this fitness advantage is mediated by a unique rhamnogalacturonan-II degradome (GH137, b-l-arabinofuranosidase; GH138, a-galacturonidase; GH139, a-2-O-methyl-l-fucosidase; GH141, a-l-fucosidase), targeting one of the most complex glycans in the cell walls of higher plants.^56^ Furthermore, *P. intestinalis* exclusively carries four polysaccharide utilization loci (PULs), a CAZymes-aggregated working unit, among which, PUL11, and PUL8 containing tandem repeat *susC*/*susD* pairs tended to be responsible for (arabino) xylans catabolism. Collectively, this evidence establishes that the colonization success and ecological impact of *Prevotella* are direct expressions of its genetic repertoire for carbohydrate metabolism. Critically, the diverse array of bioactive metabolites (e.g., SCFAs, succinate) generated from this substrate-to-product conversion serves as the primary signals and effectors that coordinate its multifaceted reinforcement of the intestinal barrier. Having established this causal link from genes to metabolites, we therefore proceed to examine how these microbial products specifically orchestrate barrier function across its physical, chemical, immune, and biological dimensions.

## *Prevotella*/*Segatella* in intestinal barrier function by a multi-layered strategy

### Physical barrier function

**Figure 3.**
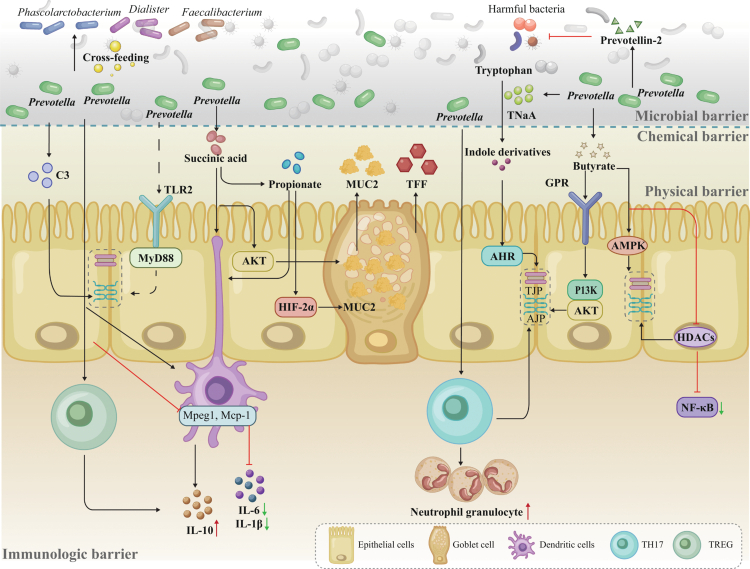
*Prevotella* and associated gut microbes regulate the intestinal barrier via microbial, chemical, physical, and immunological pathways. Their metabolites promote mucus secretion, epithelial junction integrity, and balanced immune responses, contributing to mucosal homeostasis and protection against pathogens. Abbreviation: C3, Complement Component 3; TLR2, Toll-like receptor 2; MyD88, primary response protein 88; Mpeg1, Macrophage-expressed gene 1 protein; Mcp-1, Monocyte chemoattractant protein-1; IL-10, Interleukin 10; IL-6, Interleukin 6; IL-1β, Interleukin 1 beta; TH17, T helper 17 cell; AKT, Protein kinase; BHIF-2α, Hypoxia-inducible factor 2 alpha; AHR, Aryl hydrocarbon receptor; MAPK, Mitogen-activated protein kinase; PI3K, phosphoinositide 3-kinase; TJP, Tight junction protein; AJP, Adherens junction protein; MUC2, Mucin 2; TFF, Trefoil Factor Family; HDACs, Histone deacetylases; NF-κB, Nuclear Factor-kappa B; GPR, G protein-coupled receptor; TNaA, Tryptophanase.

The gut's physical barrier fulfills various biological functions for the host, primarily blocking noxious microbiomes and regulating nutrient exchange. The tightness was maintained by junction complexes, including TJs (composed of Occludins, Claudins, Cryptokinin-1, and Zonula Occludens proteins [ZOs]), AJs, and desmosomes.^71^^,^^72^ Early recognition of microbial influences in TJs has focused on those well-known *Lactobacilli* and *Bifidobacteria*, but there is increasing interest swift to *Prevotella* or *Segatella* as its frequent dominance in the gut and evidence of intestinal barrier enhancement.^34,73‐76^ *Prevotella* upregulated the expression of tight junction proteins, including ZO-1 and Occludins, by restoring the normal expression of farnesoid X receptor (FXR), fibroblast growth factor 15 (FGF15), and Takeda G protein-coupled receptor 5 (TGR5) in the ileum, thereby strengthening the epithelial barrier.^77^ Numerous studies have further demonstrated that barrier enhancement is mediated through the activation of canonical pathways, including PI3K/Akt,^78^ AMPK,^79^ and HDAC inhibition,^80^^,^^81^ by microbial metabolites such as butyrate; the mechanisms of which are elaborated later. Speculatively, the stimulation of TJs expression is through the ligation with the toll-like receptors, specifically Toll-like receptor 2 (TLR2) on the enterocytes' apical membrane (Figure 3). This signal was transmitted intracellularly by the primary response protein 88 (MyD88) adapter and its downstream protein kinase C (PKC) pathway, ultimately helping to restore the expression and localization of TJs (Figure 3).^82^ Additionally, enhancing TJ closure via TLR2 stimulation reduces apoptosis in intestinal epithelial cells. Although direct evidence is lacking, *Prevotella* species such as *P. melaninogenica*, which express various lipoproteins like other commensals, could potentially bind to TLR2 and initiate signal transduction across the membrane.^83^

Furthermore, *P. intermedia* secretes tryptophanase (TNaA), which catalyzes the conversion of tryptophan into indole. Indole, a known ligand for the aromatic hydrocarbon receptor (AhR),^84^ has been implicated in the regulation of genes associated with tight junctions and actin cytoskeleton organization^85^ (Figure 3). This suggests that the TNaA-derived indole may contribute to the maintenance of apical junction complex integrity by modulating key actin-binding proteins, such as myosin IIA and ezrin.^85‐89^ However, caution is warranted as excessive intestinal indole can overinhibit CYP11A1, thereby compromising gut barrier integrity, while its hepatic metabolite indoxyl sulfate (IS) contributes to systemic toxicity.^90^^,^^91^ Generally, most microbially derived indole derivatives, such as indole-3-acetic acid,^92^ indole-3-lactate,^93^ indole-3-propionic acid,^94^ and indole-3-carboxaldehyde,^95^ enhance intestinal barrier function at appropriate physiological concentrations.

Butyrate, a key metabolic product of *Prevotella*, contributes to regulating TJs expression through various barrier function-associated signaling pathways. For instance, it can trigger the phosphoinositide 3-kinase (PI3K)/protein kinase B (Akt) pathway by ligating G-protein-coupled receptor (GPR) 41 and GPR43 expressed in intestinal epithelial cells (Figure 3).^96‐98^ Besides, it elevates the energetic metabolism of epithelial cells via the mediating AMP-activated protein kinase (AMPK) pathway, which upregulates the densities and distribution of Occludin and claudin (Figure 3).^79^ In addition, butyric acid, as an inhibitor of histone deacetylases (HDACs), promotes ZO-1 and Occludin production epigenetically by preventing the energy dissipation caused by nuclear factor-kappa B (NF-κB) induced proinflammation, thereby indirectly protecting the intestinal barrier (Figure 3).^80^^,^^81^ *Prevotella*-derived energy regulation was evidenced earlier that certain of them display unique metabolic adaptations: *P. bryantii* helps balance the intracellular pH by increasing the activity of proton pump ATPase in a SCFAs-condensed environment. Similarly, dominant gut commensals like *P. copri*, which colonize the mildly acidic colon, employ the same mechanisms such as proton-pumping ATPase complexes to maintain intracellular pH homeostasis.^99^ This adaptation not only ensures their survival but also reinforces the acidic microenvironment through continuous metabolic activity, and the resulting low-pH niche selectively inhibits pH-sensitive pathogens, thereby strengthening colonization resistance and promoting microbial ecological stability.

### Chemical barrier via mucin and antimicrobial peptides

Extensive studies have demonstrated that breaches in the intestinal chemical barrier are implicated in the pathogenesis of gut-related diseases, including inflammatory bowel disease (IBD)^98^ and Irritable Bowel Syndrome.^100^ The intestinal chemical barrier primarily consists of a mucus layer enriched with mucins (e.g., MUC2) and antimicrobial peptides, along with digestive juices, bile acids, and short-chain fatty acids, serving dual protective functions: physically blocks pathogen invasion and sustains commensal microbiota by providing ecological niches (Figure 3).^101^

For instance, butyrate increases MUC2 mRNA expression in goblet cells and induces the expression of trefoil factor family (TFF) peptides, thereby maintaining epithelial integrity, promoting mucosal repair, and preventing bacterial translocation (Figure 3).^22^^,^^102^ The protective effects of *Prevotella*-derived mucosa reconstitution are further exemplified in disease models: oral administration of *P. histicola* to ovariectomized (OVX) mice restored intestinal MUC2 levels and improved mucosal barrier function.^103^ In a separate ethanol-injury model, *P. histicola* also attenuated ethanol-induced gastric mucosal lesions.^75,103‐105^ Besides, *Prevotella* is commonly linked to propionate formation via the succinate pathway, in which succinate serves as a key intermediate.^106^ Propionate has been shown to activate hypoxia-inducible factor-2alpha (HIF-2α) in goblet cells through a *β*-oxidation-like metabolism, thereby upregulating MUC2 expression.^40^^,^^106^ Notably, beyond its role as a precursor, succinate itself can reportedly stimulate the AKT pathway to promote mucin secretion (Figure 3).^107^ Furthermore, *Prevotella* directly enhances mucosal immunity by producing antimicrobial peptides such as Prevotellin-2, which effectively inhibits pathogens like *Salmonella enterica* (Figure 3).^24^ Thus, *Prevotella* can promote the development and maintenance of the chemical barrier in the gut, providing a healthy eco-environment for host-bacteria crosstalk.

From an ecological perspective, *Prevotella* plays a crucial role in regulating mucin dynamics within the complex gut microbiota by counterbalancing the potential mucolytic disruptions caused by specialized commensals such as *Bacteroides thetaiotaomicron* and *Akkermansia muciniphila.*^108‐110^ Specifically, *A. muciniphila* colonizes the cecal outer mucus layer and relies heavily on mucin degradation for nutrient acquisition. *B. thetaiotaomicron*, inhabiting both the luminal and outer mucus layers, exhibits broad glycan-foraging versatility, degrading mucin, chondroitin sulfate, and hyaluronic acid, particularly during periods of low dietary fiber intake. While this adaptability offers metabolic flexibility, it may also compromise mucosal integrity when dietary fiber is scarce.^111^ In contrast, *Prevotella* species function as long-term luminal specialists that preferentially metabolize dietary plant polysaccharides rather than host mucin, independent of fiber availability. This ecological niche separation extends to their distinct immunomodulatory patterns: *A. muciniphila* is recognized for promoting local mucosal tolerance, largely through IL-10 induction,^112‐114^ whereas *Prevotella* metabolites not only stimulate mucin production but also elicit broader, more systemic anti-inflammatory responses, primarily attributed to signaling through GPR-dependent SCFA pathways.^96‐98^ Consequently, by providing a non-mucolytic, fiber-driven metabolic niche and generating unique immunomodulatory signals that modulate mucin dynamics, *Prevotella* may ecologically counterbalance the mucolytic activities of specialists like *A. muciniphila* and *B. thetaiotaomicron*, suggesting that it acts as a pivotal barrier-protective taxon.

### Immune function

The gut contains the largest portion of the human immune system, with approximately 70% of immune cells located there, playing a crucial role in maintaining intestinal homeostasis.^115^ Such robust gut lymphoid tissues are histologically diversified, and their maturating processes rely significantly on the “educator” roles of ubiquitous microbiotas in the gut. Microbial dysbiosis substantially hampers the enterocytes' immune functions, such as impaired antigen processing and presentation and antimicrobial functions, as indicated by a single-cell transcriptome study using GM-mice models.^116^ However, hypersensitivity can induce gut impairment and exacerbate the host's systemic inflammatory cascade. Balancing the inflammatory responses is also crucial in this dense microbiota environment, as it directs the flow of energy and nutrition. *Prevotella spp.* can modulate host immune responses through structurally diverse surface lipopolysaccharides and microbial metabolites, leading to context-dependent pro- or anti-inflammatory effects.^26,117‐120^

Succinate secreted by *Prevotella* enhances innate immunity by promoting the proliferation, functional activity, and antigen-presenting capacity of macrophages and dendritic cells, thereby bolstering both innate and adaptive immune responses.^119^^,^^121^ Another metabolite, propionate, promotes the generation of regulatory T cells (Tregs) and inhibits autoreactive T cells such as Th17, thereby restoring immune homeostasis.^119^^,^^121^^,^^122^ In addition, *Prevotella spp.* stimulates the high production of compliment 3 in the stromal cells of colonic lymphoid follicles, working with various secretory proteins, membrane receptors, and regulators contributing to the first layer of innate immunity defense.^123^^,^^124^ Furthermore, recent studies have shown that the immune-boosting effects of xylooligosaccharides (XOS) are linked to the proliferation of *Prevotella* species.^25^ Additionally, gut microbiota dominated by *Prevotella* produces higher levels of secretory immunoglobulin A (sIgA) compared to *Ruminococcus*-dominated microbiota.^125^

Nonetheless, growing evidence indicates that an elevated abundance of specific *Prevotella* species, including *P. copri*, in the gut may support immune balance by suppressing excessive inflammatory reactions. For instance, a prospective observational study by Ng et al. revealed that individuals with higher levels of *P. copri* in their gut microbiota experienced fewer adverse effects (e.g., diarrhea) after COVID-19 vaccination, pointing to its possible role in mitigating inflammation and fine-tuning immune reactivity.^126^ Additionally, oral administration of *P. copri* to Goto-Kakizaki rats improved immunologic equilibrium and intestinal barrier function, as evidenced by the downregulation of IL-6 and upregulation of IL-10 cytokines, alongside enhanced expression of FXR, which is associated with suppressed mucosal damage induced by excessive bacterial exposure (Figure 3).^127^ Moreover, *P. copri* can shape inflammatory chemokine responses in vivo by promoting cooperative anti-inflammatory effects within microbial consortia; in a PSC mouse model, co-intervention involving *P. copri* led to reduced hepatic Macrophage Expressed (Mpeg1) and Monocyte chemoattractant protein-1 (Mcp-1) expression (Figure 3).^128^ Mpeg1 is a marker gene that encodes macrophage-specific proteins, serving as an indicator of macrophage presence and relative abundance, whereas MCP-1 functions as a chemokine, promoting monocyte recruitment to sites of inflammation and tumors, thereby accelerating tumor progression.^129^ These immunosuppressive effects of *P. copri* are thought to be linked to its surface lipopolysaccharides (LPS):^118^ certain *P. copri*-derived LPS decreased the sensitivity of immune responses in primary human peripheral blood mononuclear cells (PBMCs), as indicated by the reduced production of pro-inflammatory cytokines, including NF-κB, tumor necrosis factor-*α* (TNF-*α*), IL-1β, and IL-6.^26^ It is important to note that LPS derived from a few members of the *Bacteroidales* family exhibit anti-inflammatory properties by suppressing TLR4-mediated immune activation.^118^

Th17 cells play a crucial role in enhancing the barrier function of intestinal epithelial cells. They promote the production of antimicrobial peptides and stimulate neutrophil aggregation by secreting cytokines such as IL-17. This process strengthens the defense capabilities of the intestinal mucosa, improving overall intestinal immunity. *P. copri* and *P. melaninogenica* can both induce Th17 cells in the gut. *P. melaninogenica* potentially balance the pro-inflammatory effects concomitantly by up-regulating IL-10.^130^ Similarly, another species *P. histicola* can also enhance the proliferation of Treg cells and dendritic cells in the lamina propria, leading to increased production of IL-10, which sequentially induces the differentiation of T cells into Tregs.^131^ Notably, *P. histicola* administrations suppress cytokine in the jejunum, colon, and ileum but give rise to their increased expressions in the duodenum, suggesting that the immune modulation through *P. histicola* is gut segment dependent. Certain species of *Prevotella* show promise as agents for promoting immune tolerance.^123^

However, evidence also highlights the strain-specific and context-dependent pathological effects within the *Prevotella* genus. For instance, *Prevotella hominis* is selectively enriched in the gut microbiota of hypothyroidism patients and exhibits strong positive correlations with systemic inflammatory markers, including C-reactive protein and TNF-*α*, suggesting its contributory role in endocrine-associated inflammation.^132^ Furthermore, while specific *P. copri* isolates may exert immunosuppressive functions, phylogenetically distinct clades of *P. copri* directly activate NF-κB signaling through LPS-TLR4 interactions, mechanistically driving vascular calcification in metabolic dysregulation.^133^ Moreover, certain *Prevotella* species in extra-intestinal niches exhibit pathogenicity: *P. oris* facilitates *P. gingivalis* colonization and accelerates periodontitis,^134^ while *P. intermedia* dysregulates arachidonic acid metabolism in severe periodontitis.^135^ Additionally, *P. bivia* promotes vaginal inflammation and recruits CCR5^+^ Th cells, potentially elevating HIV susceptibility.^117^ This collectively suggests that *Prevotella*'s immunopathological responses are determined by a complex interplay of bacterial phylogeny, host immune status, and microenvironmental cues.

### Bacteria-bacteria network

Most microbes in the gut participate in cross-feeding, forming a robust trophic network, which is also considered a biological barrier, protecting the host from pathogens.^136^ *Prevotella* is a key node in the gut microbial network, significantly influencing the stability and resilience of the ecosystem. As a pioneer fiber degrader, it degrades outer heteropolymers and produces metabolites that nourish other cross-fed members.^73^^,^^137^ *Prevotella spp*. are prominent succinate producers in the gut.^60^^,^^99^^,^^138^ Succinate can be cross-fed to succinate-utilizing taxa, including *Phascolarctobacterium* and *Dialister*, which convert succinate to propionate (Figure 3).^138‐142^ In parallel, major butyrate producers such as *Faecalibacterium* and *Roseburia* typically rely on acetate cross-feeding, with butyrate formation commonly proceeding via butyryl-CoA:acetate CoA transferase (Figure 3).^143^^,^^144^ Therefore, *Prevotella spp*. engages in synergistic interactions with other gut bacteria to boost the synthesis of SCFAs, which contribute to a reduction in intestinal pH and reinforce epithelial barrier integrity by upregulating tight junction proteins.^79^^,^^119^

*Bacillus coagulans MZY531* alleviates intestinal mucosal injury and enriches *Prevotella* and *Bifidobacterium*. *Prevotella* expansion may reshape SCFA-related cross-feeding, warranting mechanistic validation.^145^^,^^146^ Importantly, co-occurrence analyzes suggest that *Prevotella* may influence the composition of commensals in humans, including *Streptococcus*, *Enterococcus*, *Desulfovibrio*, and *Lachnospiraceae*, with potential implications for gut health.^147^ It enhances the therapeutic effect of *Lactobacillus murinus* in primary sclerosing cholangitis by increasing its relative abundance, and the combined intervention of these two microbes effectively reduces inflammation and fibrosis by inhibiting the transforming growth factor beta 1/SMAD signaling pathway.^128^ In colorectal cancer patients, depletion of *P. copri* co-occurs with depletion of *Faecalibacterium prausnitzii*, a major butyrate producer, consistent with an erosion of anti-inflammatory potential in the microbiome.^148^ Additionally, *Prevotella* is able to collaborate with other commensal bacteria to raise levels of luminal complement 3, which fortifies the gut barrier and reduces susceptibility to diarrheal diseases.^124^ Moreover, studies have shown that oral administration of *P. histicola* MCI 001 promotes the growth of *Allobaculum* by increasing the utilization efficiency of carbohydrates and acetate. This remodeling of microbial community structure and the restoration of associated metabolites, such as butyrate, contribute to reestablishing intestinal immune homeostasis.^104^

In addition, *Prevotella* utilizes type VI secretion systems (T6SS) to actively compete in the gut niche. The GA2-type T6SS (Type VI Secretion System), which is highly prevalent among the *Prevotella* genus, delivers effector toxins into prokaryotic target cells, triggering their lysis and subsequent elimination. With this mechanism, *Prevotella* can suppress the growth of competing microorganisms, boost its own population, and alter the gut bacterial composition.^149^

In summary, *Prevotella* and *Segatella* employ a multi-layered strategy, spanning physical, chemical, immune, and microbial networks to maintain and reinforce the intestinal barrier, underscoring their role as keystone symbionts. Despite their recognized ecological significance, these taxa remain largely refractory to cultivation, presenting a major obstacle to their development as strain-based therapeutics. This translational gap, as outlined in the introduction, stems firstly from a critical scarcity of culturable isolates. The very functional richness described above remains largely inferred from genomic data, lacking validation and exploitation through pure culture studies. Therefore, the following sections shift focus to the methodologies, both conventional and cutting-edge, that are essential to bring these pivotal bacteria from ecological concept to laboratory reality.

## The current practice of cultivating *Prevotella/Segatella*

To date, obtaining pure strains of *Prevotella* or *Segatella* primarily depends on traditional, labor-intensive culture methods and tailored culture conditions. We systematically reviewed culture-based studies of *Prevotella/Segatella*, summarizing the conventional isolation and cultivation practices reported in Table 1, and synthesizing a method-selection framework that spans targeted enrichment, and next-generation genomics-/data-driven recovery and identification strategies in Table 2. Generally, recovery of these strains typically requires rich basal medium, including Brain Heart Infusion agar,^76^^,^^150^^,^^151^ Schaedler agar,^152^ blood agar,^31^^,^^153^ modified peptone yeast glucose medium,^154^ etc. As obligate anaerobes, most species require strict anaerobic conditions, although limited evidence suggests possible transient aerobic survival in polymicrobial cultures (Table 2).^155^ Their growth optimization further depends on CO₂ or bicarbonate availability. For example, *P. copri* exhibits strong dependence on a bicarbonate-rich environment, with growth limitation observed at concentrations below 20 mM. Sodium ion concentration critically influences the growth kinetics, with optimal proliferation observed at 10 mM Na⁺ and significant inhibition occurring at elevated concentrations.^99^ High salinity (>6.5% NaCl) and elevated bile salts (>20% wt/vol) have been shown to suppress *Prevotella* growth.^31^ While these lineages exhibit a surprising temperature tolerance, adherence to the parental environment remains crucial for optimal growth and stability.^31^ Pure *Prevotella* colonies were isolated from fecal samples of healthy Japanese men, grown at 37℃ in a 100% CO₂ atmosphere on Eggerth Gagnon agar supplemented with 5% (v/v) horse blood.^153^ Another critical yet frequently neglected consideration is the extended cultivation: *Prevotella* typically necessitates a prolonged incubation period of 48–96 hours to resile and thrive (Table 1).^150^^,^^152,^^156‐160^

**Table 1.** Summary of cultivation conditions and corresponding isolation culture media for *Prevotella/Segatella* from published studies.

No.	Cultivation method	Culture medium	Isolated strains	Source	References
1	Fecal samples heat- or chloroform-treated, serially diluted (10⁻³–10⁻⁵), plated and anaerobically incubated at 37℃ for 96 h (85% N₂, 10% CO₂, 5% H₂); 150 colonies isolated at 48–96 h based on morphology	mBHI with 0.5 µg/mL ciprofloxacin; mBHI with 1 µg/mL gentamycin	*P. copri*; *P. stercorea*	Human fecal samples	[156]
2	Human fecal samples plated anaerobically at 37℃ for 48–72 h; colonies selected based on yellow-ring morphology	BHI blood agar (5% sheep blood), xylan-supplemented (5%), or inulin-supplemented (5%) media	*P. copri*	Human fecal samples	[76]
3	Isolation from conventional sow feces under anaerobic conditions (80% N₂, 20% CO₂)	PYG medium	*P. copri*	Conventional sow feces	[154]
4	Anaerobic plating of fecal samples at 37℃ for 48–72 h; identification confirmed by colony PCR and 16S rRNA sequencing	BHI Blood Agar with Vancomycin	*P. copri*	Human fecal samples	[151]
5	Clinical samples subcultured and incubated anaerobically at 37℃ for 2–5 d; *Prevotella* isolates further cultured on additional media	Selective anaerobic blood agar (nalidixic acid/vancomycin)	*P. histicola*; *P. nigrescens*	Respiratory samples (cystic fibrosis/chronic obstructive pulmonary disease patients)	[150]
6	Samples were streaked using 10 μl loops and incubated anaerobically with GasPak™ EZ at 37℃ for 3–5 d (up to 1 week when required) to obtain isolated colonies	Schaedler agar enriched with kanamycin/vancomycin	*P. melaninogenica*; *P. histicola*; *P. salivae*; *P. veroralis*; *P. maculosa*; *P. nanceiensis*	Saliva samples	[152]
7		Veillonella agar base +/- vancomycin M416	*P. histicola*; *P. veroralis*; *P. melaninogenica*; *P. nanceiensis*		
8		GC–Lect	*P. melaninogenica*; *P. nanceiensis*; *P. loescheii*		
9		Brucella Agar	*P. melaninogenica*; *P. veroralis*; *P. nanceiensis*		
10		non-selective Schaedler agar	*P. histicola*; *P. melaninogenica*		
11		Tryptic soy agar with sheep blood	*P. melaninogenica*; *P. veroralis*		
12	Clinical swabs reconstituted in 20% NYCB with 2% horse serum in 0.9% saline; plated and cultured anaerobically at 37℃ for 72 h using gas-generating bags with oxygen indicator	GSA blood agar	*P. bivia*	Dry vaginal swabs	[159]
13	Bacterial isolates from sputum cultured anaerobically on three media types at 37℃ for 21 d; McFarland suspensions in saline were inoculated onto enriched agar plates for testing	Brucella blood agar — with 5% sheep blood, vitamin K₁, and hemin	*P. melaninogenica*	Sputum from patients with cystic fibrosis	[161]
14	Fecal samples transported at 5℃ under anaerobic conditions, homogenized in pre-reduced PBS, serially diluted (10^−1^–10^−10^), and plated; cultures incubated at 37℃ under both aerobic and anaerobic conditions after 1.5 h oxygen exposure	MacConkey agar, TSA, XLD agar, Nutrient agar, and Blood agar (aerobic/anaerobic); diluted with pre-reduced	*P. copri*	Human fecal samples	[162]
15	Rumen fluid processed anaerobically, serially diluted, and cultured in roll tubes at 37℃ for 48–72 h	Modified YTR agar — with yeast extract, peptone, rumen fluid, glucose, cellobiose, redox agents (resazurin/cysteine), hemin, and 1.2% agar	*Prevotella* OTU218	Holstein cow rumen fluid sample	[163]
16	Bacterial isolates subcultured and incubated anaerobically at 37℃ for 48 h (80% N₂, 10% CO₂, 10% H₂) using anaerobic jars or cabinets	Brucella blood agar supplemented with hemin (5 mg/L) and vitamin K (1 mg/L)	*P. melaninogenica*; *P. bivia*; *P. buccae*	Human clinical specimens	[160]
17	Bacterial strains cultured under anaerobic conditions at 37℃	Tryptic soy agar with 5% sheep blood	*P. bivia*; *P. buccae*; *P. corpori*; *P. denticol*; *P. disiens*; *P. histicola*; *P. timonensis*	BEI or ATCC	[164]
18	Incubated at 37℃ for 48 h under 100% CO₂	Eggerth Gagnon agar with 5% horse blood Eggerth Gagnon	*P.copri*; *P. stercorea*	DSMZ	[153]
19	Cultured anaerobically at 37℃ for 48 h using Anaerocult™ A system	Columbia blood agar (CBA) — Columbia base agar with 5% defibrinated horse blood	*P. bivia* ATCC 29303^T^	ATCC	[158]
20	*P. copri* DSM 18205 cultured anaerobically at 37℃ under N₂/CO₂ (80/20%); growth monitored by OD₆₀₀ nm	Modified PYG Medium	*P. copri* DSM 18205	The German Collection of Microorganisms and Cell Cultures	[99]
21	Bacterial strains cultured from cryopreserved stocks at 37℃, followed by subculture in fresh BHI broth to logarithmic phase (OD₆₀₀ = 0.3–0.5)	Enriched BHI broth/agar — supplemented with vitamin K3, hemin, and L-cysteine	*P. copri* DSM 18205	The German Collection of Microorganisms and Cell Cultures	[24]
22	Type and isolated *Prevotella* strains maintained on slant agar and cultured in modified medium for growth analysis	Modified YTR slant agar	*P. ruminicola* B23^T^ (JCM 8259); *P. bryantii* B_1_4^T^ (DSM 11371)	Obtained commercially	[157]
23	Incubated anaerobically for 24 h at 37℃ using the AnaeroGen Atmosphere Generation system	New York City III broth— containing proteose peptone, glucose, HEPES, NaCl, yeast extract, and 10% heat-inactivated horse serum	*P. bivia* ATCC 29303 ^T^	ATCC	[165]

For the targeted isolation and enrichment of *Prevotella*, current strategies primarily rely on substrate-specific cultivation, such as supplementing media with carbohydrates like xylan,^76^ isomaltooligosaccharides (IMOs), inulin, or starch,^30^ combined with corresponding indicators for visual identification. Fecal-derived *Prevotella* (e.g., *P. copri*) can be enriched/isolated on xylan-based selective media supplemented with a pH indicator (bromocresol purple), enabling visual recognition via a purple-to-yellow color shift after ~48 h of anaerobic incubation.^57^ Strains such as *S. bryantii* TF1-3 demonstrate robust growth in media enriched with complex glycans like starch and galactomannan.^166^ Additionally, cross-feeding strategies involving metabolites derived from *Fusobacterium nucleatum* have successfully recovered *Prevotella*, evident as distinct growth zones in agar.^167^ However, these conventional strategies are often limited by low throughput, labor intensity, and the imprecise supplementation of substrates without genomic guidance. These constraints thus create a compelling rationale for the development of advanced cultivation strategies that integrate high-throughput capacity with precision-targeted enrichment of *Prevotella* and *Segatella* (Table 2).

## Next-generation cultivation: a genomics- and data-driven pipeline for *Prevotella* and *Segatella*

### Genomics-guided precise cultivation and screening

Theoretically, the *in vitro* cultivation of any bacterium is achievable once its precise nutritional and physiological requirements are satisfied. In practice, however, defining these parameters a priori remains a principal bottleneck, especially for the vast diversity of uncultured lineages revealed by high-throughput sequencing, for which phenotypic data are absent. The expansion of genomic sequencing has catalyzed the development of cultivation strategies grounded in sequence-derived predictions. Early computational tools, exemplified by the Komodo module of the ModelSEED platform (2015),^168^ employed 16S rRNA gene sequence similarity to propose medium formulations, thereby offering a first-step guide for targeted isolation. Nevertheless, a transformative advance emerged with metagenome-assembled genomes (MAGs), which reconstruct near-complete genomic blueprints from complex environmental sequence data. MAGs provide the foundation for constructing genome-scale metabolic models (GEMs) and for delineating phylogenetically coherent populations (e.g., via ANI-based clustering). Taxa within these phylogenetically defined clusters typically share core metabolic features, enabling robust *in silico* inference of organism-specific nutrient demands and metabolic potential.^169^ Contemporary automated platforms, such as CarveMe,^170^ gapseq,^171^ and ModelSEED,^172^ systematically translate genomic information into predicted core metabolic networks, providing a rational basis for the design of tailored cultivation media. This genome-informed approach has been successfully translated into practice.^173^ In a 2024 study of a nutritional intervention in Bangladeshi children, researchers identified MAGs classified as *P. copri* whose abundance was positively associated with ponderal growth in children. Functional annotation of these MAGs pinpointed polysaccharide utilization loci (PULs) with predicted specificity for dietary glycans, including mannans and galactans. Accordingly, this genomic inference directly informed the cultivation strategy: defined media were formulated with these glycans as exclusive carbon sources, leading to the successful screening of *P. copri* strain BgF5_2. Subsequent characterization confirmed that the BgF5_2 strain's PUL complement and carbohydrate utilization profile aligned closely with the genetic repertoire of the target MAG.^174^ Moving forward, the strategy of employing genomic predictions for substrate design represents, as a precise, reliable, and direct route to recover *Prevotella*, the emerging standard practice for translating sequencing data into functional insights (Table 2).

### Genomics-guided antibiotic-based selective enrichment

Beyond predicting substrates, genomic analysis further enables the rational design of targeted enrichment strategies using antibiotics, offering a robust approach to isolate fastidious *Prevotella* from complex microbial communities. The species-specific prevalence of antibiotic resistance genes (ARGs) serves as a paradigm for genomically guided enrichment. For instance, the *β*-lactamase gene cfxA exhibits a distinct, species-specific prevalence. It is near-ubiquitous in species like *P. melaninogenica* and *P. buccalis* (100% of isolates), yet less common in others such as *P. bivia* (66.7%) or *P. nigrescens* (40%).^175^ This genomic pattern can be directly translated into a targeted cultivation approach. Similarly, the tetracycline resistance gene *tetQ* (e.g., against doxycycline) and the macrolide resistance gene *ermF* (e.g., against azithromycin) are widely distributed among *Prevotella* species, with notably high positivity rates of *tetQ* in *P. timonensis* and *ermF* in *P. buccalis.*^150^^,^^176^ This genetic distribution thus establishes a basis for genomically guided selective media. As demonstrated in early studies, tetracycline-supplemented media enabled the selective isolation of *tetQ*-harboring Prevotella strains.^177^ Combining these antibiotics can synergistically suppress broad-spectrum competitors, including *Pseudomonas aeruginosa* and *Staphylococcus aureus.*^178^^,^^179^ However, a critical consideration is that the presence of antibiotic resistance genes such as *cfxA*, *tetQ*, and *ermF*, directly contravenes the safety criteria outlined by multiple international regulatory frameworks, including the European Food Safety Authority's Qualified Presumption of Safety (QPS) assessment, the U.S. FDA's Generally Recognized as Safe (GRAS) requirements, and the FAO/WHO probiotic guidelines. These regulatory bodies uniformly emphasize the exclusion of antimicrobial resistance genes in probiotic candidates.^180^^,^^181^ Therefore, any enrichment strategy for developing *Prevotella*-based probiotics must include a secondary optimization step, such as genetic editing, to remove or modify noncompliant resistance genes, thereby mitigating horizontal gene transfer risks and ensuring regulatory compliance prior to their use as direct-fed microbials (Table 2).^182^

In practice, the broad-selection strategy leveraging intrinsic physiological resistance is more routinely employed in the initial isolation of *Prevotella*, primarily because it effectively suppresses fast-growing, non-target competitors without requiring prior genomic knowledge of the specific strain.^169^ A cornerstone is the use of kanamycin–vancomycin selective media (e.g., LKV/KVLB), which suppress many Gram-positive and facultative competitors and thereby enrich obligate anaerobic Gram-negative bacilli such as *Prevotella* spp., and is routinely used in anaerobic culture workflows (Table 2).^183‐185^ For instance, Li et al. successfully isolated *P. copri* strains using vancomycin-supplemented BHI blood agar,^151^ and Hammad et al. obtained pure isolates of *P. histicola*, *P. salivae*, *P. veroralis*, *P. maculosa*, and *P. nanceiensis* with vancomycin in Schaedler agar.^152^ While not as precise as genomic substrate profiling, antibiotic strategies remain essential for isolating *Prevotella* and *Segatella* by suppressing fast-growing competitors, yet their application must be carefully optimized to balance efficacy with genomic safety for downstream viability.

### High-throughput targeted strain recovery and identification

A prime example is the automated anaerobic microbial isolation and cultivation system (CAMII) developed by Huang et al. This system integrates image-based morphological analysis, which captures key traits such as size, shape, color, texture, and edge definition, with genomic data to establish a data-driven, targeted colony-picking platform. The CAMII platform enabled the successful recovery of 26,997 isolates, which represented over 80% of all abundant microbial taxa in the samples. This achievement provides compelling evidence that the majority of gut microorganisms can be cultured *in vitro*. Within the publicly accessible CAMII platform (http://microbial-culturomics.com/), 50 *Prevotella* isolates, including species such as *P. corporis*, *P. jejuni*, *P. melaninogenica*, *P. saccharolytica*, *P. histicola*, *P. veroralis*, and *P. copri* were systematically isolated based on colony morphology (Table 2).^186^ This study validated the feasibility of morphology-guided, AI-assisted targeted isolation and greatly encouraged functional investigations into rare and fastidious bacteria. Beyond morphology-based selection, strategies relying on optical signatures or specific molecular features offer complementary approaches within high-throughput workflows of targeting *Prevotella*. Ultraviolet (UV) labeling leverages the distinctive brick-red fluorescence of *Prevotella* colonies under UV light as a rapid phenotypic marker for differentiation (Table 2).^152^ Fluorescence-activated cell sorting (FACS) is a flow cytometry-based technique. It uses rRNA-directed probes alongside light scattering and fluorescence parameters (e.g., FL1 for FITC, FL6 for Cy5) to physically isolate target cells, including previously uncultured *Prevotella*, directly from fecal samples (Table 2).^187^

By contrast, the more widely adopted and flexible approach for high-throughput identification is the molecular fingerprinting technique of matrix-assisted laser desorption/ionization time-of-flight mass spectrometry (MALDI-TOF MS) (Table 2). This approach utilizes established reference spectral databases for rapid taxonomic classification (e.g., 96 isolates per run), which subsequently guides and streamlines targeted genetic verification of strains. This method has demonstrated remarkable accuracy in discriminating closely related *Prevotella* species, such as *P. intermedia* and *P. nigrescens*, which are conventionally indistinguishable. Demonstrating 88.6% species-level accuracy, it significantly outperforms traditional biochemical tests that exhibit misidentification rates as high as 70.7%.^188^ Its clinical utility is further reinforced by multi-center studies using systems such as MALDI Biotyper and VITEK MS, with reported species-level identification rates of 83.1% across diverse geographic isolates.^189^^,^^190^ However, its performance is highly dependent on the comprehensiveness of the spectral database. Underrepresented species such as *P. aurantiaca* and *P. jejuni* are frequently misidentified, underscoring the necessity for continuous expansion of reference libraries.^191^

Other advanced technologies also demonstrate considerable potential for targeted bacterial isolation and could be adapted for future *Prevotella* cultivation. For instance, microfluidics technology circumvents inter-strain competition by physically confining individual cells within engineered microdroplets or microchannels, enabling single-cell cultivation.^192^ This approach can be effectively integrated with the selective media previously established to significantly enhance the capture efficiency of *Prevotella* (Table 2).^193^^,^^194^ Furthermore, a magnetic nanoparticle-based in-situ culture strategy utilizes substrate-coated magnetic nanoparticles to directly enrich target bacteria from complex samples, a process that significantly streamlines isolation while better preserving the original physiological states of the bacteria (Table 2).^195‐197^

Collectively, although this genomics- and data-driven pipeline faces challenges in cost and accessibility, the integration of these advanced cultivation technologies, from automated robotics to microfluidics and precise substrates, is systematically overcoming the isolation bottlenecks for *Prevotella* and *Segatella*, directly advancing their development into safe and applicable direct-fed microbials.

## Toward future precision and safety in *Prevotella*/*Segatella* therapeutics

However, translating these cultivated strains into safe and effective therapeutics presents a distinct challenge: their ecological functions and clinical impacts are highly strain- and context-dependent,^37^ as systematically categorized in Table 3. For instance, clinical observations reveal that the elevated abundance of specific *Prevotella* species (e.g., *P. stercorea*) correlates with disease susceptibility and severity in Spinal Arthritis.^198^^,^^199^ Similarly, while *P. copri* generally benefits gut health under fiber-rich conditions; its excessive succinate production may paradoxically activate pro-inflammatory genes (*Ccr7, Nos2*) and exacerbate arthritis.^200^^,^^201^ Additionally, intestinal overgrowth of *Prevotella* has been implicated in metabolic dysfunction-associated fatty liver disease (MAFLD), potentially mediated through bacterial components such as LPS.^202^ Notably, the genus's ecological impact extends beyond the gastrointestinal tract. The gut commensal *P. bivia* has been mechanistically linked to the disruption of vaginal microbiota. This translocation and subsequent dysbiosis are hypothesized to elevate the risk of preterm labor and other adverse pregnancy outcomes.^164^ These findings underscore that accurate assessment at the species level, while essential, remains insufficient for guiding safe clinical practice.

**Table 2.** A method-selection framework for cultivating, enriching, and identifying *Prevotella*/*Segatella* spp.: conventional workflows and next-generation genomics-/data-driven strategies summarized from published studies.

No.	Technique	Key implementation (as used/described)	What it adds	References
**Genomics- and data-guided cultivation**				
1	MAG/GEM-guided medium design	Sequence-based medium prediction (Komodo/ModelSEED) + GEM tools (CarveMe, gapseq) from 16S/MAGs; formulate defined media with inferred substrates/glycans.	Translates sequencing into actionable cultivation recipes.	[168‐174]
Selective enrichment and isolation				
2	ARG-informed antibiotic selection	Use ARG prevalence (*cfxA*/*tetQ*/*ermF*) to select beta-lactams/tetracyclines/macrolides for targeted enrichment; add safety/governance for therapeutic use.	Improves selectivity; highlights ARG safety constraints.	[150,175‐179]
3	Broad selective media (KVLB/LKV; vancomycin workflows)	KVLB/LKV or vancomycin-supplemented BHI/Schaedler to suppress Gram-positive/facultative competitors and enrich obligate anaerobic Gram-negative bacilli.	Practical first-pass enrichment without prior genomics.	[151,152] [183‐185]
High-throughput recovery and identification				
4	AI-guided robotic colony picking (CAMII)	Colony imaging + ML ranking + robotic picking under anaerobiosis; integrate with genomic confirmation.	Automated, label-free, scalable isolation.	[186]
5	UV fluorescence screening	UV inspection; prioritize brick-red fluorescent colonies for confirmation.	Rapid phenotypic marker for colony selection.	[152]
6	FACS with rRNA probes	rRNA-probe labeling + flow sorting; cultivate sorted fractions anaerobically.	High-specificity recovery from mixed communities.	[187]
7	MALDI-TOF MS	MALDI Biotyper/VITEK MS protein fingerprints for rapid ID; sequence-confirm ambiguous calls; expand spectral libraries.	Rapid, scalable ID; database coverage is limiting.	[188‐191]
Emerging platforms				
8	Microfluidics (single-cell cultivation)	Single-cell confinement in droplets/microchannels; pair with selective media/substrates; parallel screening.	Reduces competition; increases capture throughput.	[192‐194]
9	Magnetic nanoparticle-based in situ enrichment	Substrate-coated magnetic nanoparticles enrich targets from complex samples; magnetic retrieval followed by anaerobic cultivation.	Streamlined enrichment; preserves physiological state.	[195‐197]

Future therapeutic applications of *Prevotella* and *Segatella* in gut health management must be grounded in a strain-specific and host-aware precision framework. The clinical suitability of a given strain should be determined by its functional gene repertoire, complemented by pre-intervention assessment of host factors. For instance, *P. copri* strains carrying glycoside hydrolase and glycosyltransferase genes may serve as candidate therapeutics for metabolic syndromes due to their potential to enhance glucose metabolism and modulate microbiota composition.^76^ Similarly, the therapeutic potential of *P. histicola* in inflammatory bowel disease relies on genetic determinants associated with enhanced tight junction expression and GAPDH/IL-17RB signaling, which collectively reinforce epithelial barrier integrity and mitigate inflammatory cascades.^75^ In contrast, *P. copri* strains encoding peptidylarginine deiminase must be strictly contraindicated in patients with rheumatoid arthritis due to their pathogenic potential to exacerbate joint inflammation via protein citrullination and TLR4/NF-κB-dependent pathways.^199,203‐205^ Thus, high-resolution genomic profiling, coupled with host-factor stratification, will be indispensable for translating the functional diversity of *Prevotella* and *Segatella* into safe, effective, and personalized microbiome-based therapies. This precision approach will ultimately redefine their role in clinical practice, from ambiguous commensals to targeted therapeutic agents.

**Table 3.** Ecological and clinical profiles of *Prevotella* species.

Species name		Ecological niche	Roles in health/disease	References
***P. rectalis***		Human rectum	No direct association with human diseases reported	[206]
***P. stercorea***		Human gut	1. Supports infant intestinal development; becomes dominant post-weaning but declines in the elderly	[136]
			2. Decreases androgen levels in mice, delaying castration-resistant prostate cance onset	[207]
			3. Reduced in atopic dermatitis patients	[208]
			4. Negatively correlates with symptomatic dermographism duration	[209]
			5. Depleted in dry eye disease	[210]
***P. copri***		Human gut	1. Reduces IL-6, NF-κB, TNF-*α*, IL-1β; increases IL-10 and FXR, improving gut barrier; Mitigates COVID-19 vaccination side effects (e.g., diarrhea); suppresses Mpeg1 and Mcp-1, reducing inflammation and monocyte aggregation	[118,127,128,211]
			2. Combines with *L. murinus* to inhibit TGF-β1/Smad pathway, alleviating primary sclerosing cholangitis	[212]
			3. Alleviates sarcopenia by preserving muscle mass and function	[128]
			4. Ameliorates neurological deficits via GUO-PI3K/Akt pathway post-TBI	[73]
			5. Alleviates hyperglycemia and regulates gut microbiota	[76]
***P. histicola***		Human gut	1. Repairs intestinal barrier and regulates immunity. Alleviates multiple sclerosis and IBD	[131]
			2. Attenuates ethanol-induced gastric mucosal lesions via anti-ferroptotic System Xc-/GPX4 axis	[75]
			3. Protects from arthritis by expanding *Allobaculum* and increasing butyrate	[104]
			4. Improves psoriasis in clinical trials	[213]
			5. Reduces bone loss via gut microbiota-bone axis	[214]
			6. Improving DSS induced colitis by inhibiting the IRE1α - JNK pathway that suppresses ER stress and NF - κB signaling	[215]
			7. Depleted in high-risk chronic obstructive pulmonary disease exacerbators	[216]
***P. ruminicola***	Ruminant rumen		Supports rumen health and feed digestion; restores gut microbiota balance, reducing oxidative stress	[54]
***P. hominis***	Human gut		Linked to intestinal health; modulates host metabolism and immunity	[217]
***P. jejuni***	Human small intestine (jejunum)		Associates with celiac disease; may disrupt small intestine health	[218]
***P. lacticifex***	Rumen of cattle		No direct disease link; potentially reduces methane emissions and improves nutrient absorption in ruminants	[163]
***P. massiliensis***	Human oral cavity and gut		Generally symbiotic but implicated in bacteremia	[219]
***P. rara sp. nov.***	Human feces		Degrades host trypsin, protects intestinal IgA levels, and limits pro-inflammatory signaling	[220]
***P. communis***	Sheep rumen		No direct disease correlation reported	[221]

## Conclusion

*Prevotella* and *Segatella* hold vast, untapped therapeutic potential, yet they also embody the quintessential challenge of translating complex microbiota ecology into precision medicine. While their expansive CAZyme arsenals and sophisticated cross-feeding networks establish them as master regulators of intestinal barrier homeostasis, this very functional complexity, coupled with profound strain-level diversity and context-dependent behavior, precludes conventional probiotic development. The path forward thus demands a dual innovation: first, leveraging high-throughput, genomics-driven culturomics to transform these elusive taxa into accessible strain libraries; and second, establishing causal genotype-phenotype frameworks that bridge taxonomic diversity to predictable gut-related clinical outcomes. Ultimately, their therapeutic promise resides not in broad supplementation but in the rational redesign of microbial communities through mechanism-informed strain selection, a paradigm shift where *Prevotella* and *Segatella* evolve from ecological indicators to precisely targeted living biotherapeutics.

## Abbreviations

AAI: Amino Acid Identity
AhR: Aryl hydrocarbon receptor
AI: Artificial intelligence
AJs: Adherens junctions
Akt: Protein kinase B
AMPK: AMP-activated protein kinase
ARGs: Antibiotic resistance genes
BEI: Biodefense and Emerging Infections Research Resources Repository
BHI: Brain Heart Infusion
HIF-2α: Hypoxia-inducible factor 2 alpha
B-type: Bacteroides-dominated type
C3: Complement component 3
CAMII: Cultivation system
CAZymes: Carbohydrate-active enzymes
CBA: Columbia blood agar
CE: Carbohydrate esterases
cfxA: β-lactamase gene
DSMZ: German Collection of Microorganisms and Cell Cultures, Braunschweig
FACS: Fluorescence-activated cell sorting
FGF15: Fibroblast growth factor 15
FXR: Farnesoid X receptor
GEM: Genome‑scale metabolic models
GH: Glycoside hydrolases
GM-mice: Germ-free mice
GPR: G-protein-coupled receptor
GRAS: Generally Recognized as Safe
HDACs: Histone deacetylases
IBD: Inflammatory bowel disease
IL: Interleukin
IS: Indoxyl sulfate
IMOs: Isomaltooligosaccharides
LEEs: Large extrachromosomal elements
LPS: Lipopolysaccharides
MAGs: Metagenome-assembled genomes
MAFLD: Metabolic dysfunction-associated fatty liver disease
MALDI-TOF MS: Matrix-assisted laser desorption/ionization time-of-flight mass spectrometry
mBHI: Modified brain heart infusion
MCP-1: Monocyte chemoattractant protein-1
Mpeg1: Macrophage Expressed
MUC2: Mucin 2
MyD88: Myeloid differentiation primary response protein 88
NF-κB: Nuclear factor kappa-light-chain-enhancer of activated B cells
OVX: Ovariectomized
PBMCs: Peripheral blood mononuclear cells
PI3K: Phosphoinositide 3-kinase
PKC: Protein kinase C
PL: Polysaccharide lyases
POCP: Percentage of Conserved Proteins
P-type: *Prevotella*-dominated type
PULs: Polysaccharide utilization loci
PYG: Peptone yeast glucose medium (modified)
QPS: Qualified Presumption of Safety
SCFAs: Short-chain fatty acids
sIgA: Secretory immunoglobulin A
T6SS: Type VI secretion system
TFF: Trefoil factor family
TGR5: Takeda G protein-coupled receptor 5
Th17: T helper 17 cells
TJs: Tight junctions
TLR2: Toll-like receptor 2
TLR: Toll-like receptor
TNaA: Tryptophanase
TNF-α: Tumor necrosis factor-alpha
Tregs: Regulatory T cells
UV: Ultraviolet
XOS: Xylooligosaccharides
ZOs: Zonula occludens proteins
